# EHMTI-0242. Treating chronic tension-type headache with and without pericranial tenderness in association with a feeling of deontological guilt

**DOI:** 10.1186/1129-2377-15-S1-C19

**Published:** 2014-09-18

**Authors:** E Guertzenstein

**Affiliations:** 1 Divisão de Clínica Neurocirúrgica, Instituto de Neurologia / Hospital das Clínicas da Faculdade de Medicina da Universidade de São Paulo, São Paulo, Brazil

## 

Chronic tension type headache is a disorder evolving from frequent episodic tension-type headache, with daily or very frequent episodes of headache, typically bilateral, pressing or tightening in quality and of mild to moderate intensity, lasting hours to days, or unremitting. The pain does not worsen with routine physical activity, but may be associated with mild nausea, photophobia or phonophobia. The feeling of deontological guilt occurs in individuals who, violating moral standards of the society in which they live, wonder how they were capable of such an attitude.

In this guilt feeling brain areas that mediate disgust are stimulated as the insulas.

The proposal was to evaluate the ineffectiveness of the treatments [antidepressants, anticonvulsants, beta-blockers, NSAIDs or acetaminophen, tramadol, acetylsalicylic acid, opioids, muscle relaxants, psychotherapy, acupuncture] to which that 21 patients (15 men and 6 women) aged 40 to 52 were submitted for 2 years after stealing money from their clients motivated by revenge. All patients were professionals, no previous psychiatric and neurologic disorders. The headache started in sequence to no evidence of the crime.

Counseling replaced treatments. The pain only remitted when each patient donated the stolen amount to charitable institutions to assuage guilt.

This study presents limitations: it is retrospective with a limited number of patients, showing a specific subpopulation of chronic tension-type headache. It is important to note that the criterion was limiter. This research supports a main conclusion that brain changes can be corrected, in this case, with the recognition of error and change of conduct.

No conflict of interest.

